# Supplementary material for: Combined PK/PD Index May Be a More Appropriate PK/PD Index for Cefoperazone/Sulbactam against Acinetobacter baumannii in Patients with Hospital-Acquired Pneumonia
Source: Antibiotics (Basel). 2022 May 23;11(5):703. doi: 10.3390/antibiotics11050703 (PMC9138011; doi:10.3390/antibiotics11050703)
Supplement: Supplementary file 1 [file antibiotics-11-00703-s001.zip › antibiotics-1730254-supplementary.pdf]

Supplementary Materials

Title: Combined PK/PD Index May Be a More Appropriate PK/PD Index for Cefoperazone/Sulbactam Against *Acinetobacter baumannii* in Patients with Hospital-Acquired Pneumonia

Table S1. Distribution frequency of MIC of cefoperazone/sulbactam (%)

Bacteria (strains)	Cefoperazone/sulbactam MIC (mg/L)								
	1/0.5	2/1	4/2	8/4	16/8	32/16	64/32	128/64	256/128
A. baumannii (32)	6.3	6.3	12.5	12.5	<u>21.9</u>	18.8	**15.6**	3.1	3.1
P. aeruginosa (25)	0	8	20	<u>24</u>	32	**12**	4	0	0

Underline and bold indicate corresponding MIC are MIC_{50} and MIC_{90}, respectively

Table S2. Effect of high blood pressure on %T>MIC of cefoperazone

%T>MIC (Mean±SD)		Difference of %T>MIC
high blood pressure=0	high blood pressure =1	
84.5±25.2 (%)	85.5±25.7 (%)	1.0±1.4 (%)

Difference = %T>MIC(high blood pressure=1) - %T>MIC(high blood pressure=0). 1 means yes, 0 means no

Table S3. Effect of covariates on %T>MIC of sulbactam

	Age=18 years	Age=46 years	Age=70 years
Do not take baclofen	31.11±20.64 (%)	41.75±25.55 (%)	58.29±29.83 (%)
Taking baclofen	32.05±21.76 (%)	42.93±26.48 (%)	59.45±30.52 (%)

Results were shown as Mean±SD

Table S4. PK/PD cutoff based on PK/PD index for cefoperazone (mg/L). Dosing frequency was q8h. Target was for clinical efficacy against infection by Ab.

Based on %T>MIC (target=54.8%)

Dose	Infusion time (h)				
(g)	0.5	1	2	3	4
1	8	8	8	16	16
1.5	16	16	16	16	16
3	32	32	32	32	32
4	32	32	32	64	64
6	64	64	64	64	64

Based on AUC_{0-24}/MIC (target=44.3)

Dose (g)	1	1.5	3	4	6
PK/PD cutoff	8	8	16	32	32

Table S5. PK/PD cutoff based on PK/PD index for sulbactam (mg/L). Dosing frequency was q8h. Target was for clinical efficacy against infection by Ab.

Based on %T>MIC (target=36.6%)

Dose	Infusion time (h)				
(g)	0.5	1	2	3	4
1.5	2	2	4	8	8
2	2	4	4	8	8
3	4	4	8	16	16

Based on AUC_{0-24}/MIC (target=23.3)

Dose (g)	1.5	2	3
PK/PD cutoff	4	8	8

Table S6. Cumulative fraction of response (CFR) based on PK/PD index for cefoperazone (%). Dosing frequency was q8h. Target was for clinical efficacy against Ab.

Based on %T>MIC (target=54.8%)

Dose	Infusion time (h)				
(g)	0.5	1	2	3	4
1	56	57	60	62	64
1.5	67	68	71	73	75
2	74	76	78	80	82
3	84	85	87	89	90
4	90	90	92	93	94
6	94	95	95	96	96

Based on $AUC_{0\text{-}24}$/MIC (target=44.3)

Dose (g)	1	1.5	2	3	4	6
CFR (%)	47	59	67	78	85	92

Table S7. Cumulative fraction of response (CFR) based on PK/PD index for sulbactam (%). Dosing frequency was q8h. Target was for clinical efficacy against Ab.

Based on %T>MIC (target=36.6%)

Dose	Infusion time (h)				
(g)	0.5	1	2	3	4
1	38	42	49	56	59
1.5	48	52	60	67	70
2	55	60	68	75	78
3	66	69	78	85	87

Based on AUC_{0-24}/MIC (target=23.3)

Dose (g)	1	1.5	2	3
PK/PD cutoff	49	61	69	79

Table S8. Construction of combined PK/PD index and its correlation with clinical efficacy against Ab

Combined PK/PD index	$P_{logistic}$	P_{cross}	$P_{logistic2}$
$\%(T>MIC_{cpz}*T>MIC_{sul})$	0.047	0.020	0.041
$AUC_{0-24}/MIC_{cpz}*AUC_{0-24}/MIC_{sul}$	0.299	0.075	0.100
$Ln(AUC_{0-24}/MIC)_{cpz}*Ln(AUC_{0-24}/MIC)_{sul}$	0.057	0.030	0.057
$AUC_{0-24}/MIC_{cpz}*\%T>MIC_{sul}$	0.206	0.051	0.078
$Ln(AUC_{0-24}/MIC)_{cpz}*\%T>MIC_{sul}$	0.065	0.020	0.041
$\%T>MIC_{cpz}*AUC_{0-24}/MIC_{sul}$	0.121	0.030	0.057
$\%T>MIC_{cpz}*Ln(AUC_{0-24}/MIC)_{sul}$	0.040	0.025	0.042

$P_{logistic}$: P value obtained from logistic regression; P_{cross}: P value obtained from cross tabulation; $P_{logistic2}$: P value obtained from logistic regression after combined PK/PD index was transformed into binary data (value was 1 if it is greater than target, else it was 0)

cpz: cefoperazone; sul: sulbactam

Table S9. Cumulative fraction of response (CFR) of %($T>MIC_{cpz}$*$T>MIC_{sul}$) against Ab (%). Dosing frequency was q8h.

Regimen of cefoperazone/ sulbactam	Clinical efficacy					Microbiological efficacy				
	T=0.5	T=1	T=2	T=3	T=4	T=0.5	T=1	T=2	T=3	T=4
1g/1g	32	36	42	48	53	18	20	24	29	36
2g/1g	36	40	46	54	58	19	22	25	31	38
1.5g/1.5g	42	45	52	60	64	25	27	32	38	46
2g/2g	49	53	61	68	72	30	33	38	45	54
2g/3g	56	60	67	73	77	37	40	45	52	62
6g/1g	38	42	49	56	59	20	22	26	31	38

CFR for CPZ/SUL 3g/1g, 4g/1g was closed to that of CPZ/SUL (2g/1g), therefore it was not shown in the table

T means infusion time (h)

Table S10. Cutoff based on PTA($AUC_{0\text{-}24}/MIC_{cpz}$)*PTA($AUC_{0\text{-}24}/MIC_{sul}$) (mg/L). Dosing frequency was q8h.

Regimen of cefoperazone/sulbactam	Clinical efficacy	Microbiological efficacy
2g/1g	8	4
2g/2g	16	4
2g/3g	16	4
4g~6g/1g	8	4

Cutoff was for cefoperazone

Table S11. Comparison of PK/PD cutoff obtained from combined PTA or single drug

PK/PD index	Type of efficacy	Dosing regimen of CPZ/SUL (q12h)				
		2g/1g	2g/2g	2g/3g	4g/1g	6g/1g
%T>MIC	Clinical efficacy	=SUL (0.5-4 mg/L)	=SUL (1-8 mg/L)	Close to SUL (2-16 mg/L)	=SUL (0.5-4 mg/L)	=SUL (0.5-4 mg/L)
	Microbiological efficacy	=SUL (0.125-0.5 mg/L)	Close to SUL (0.25-2 mg/L)	Close to SUL (0.5-1 mg/L)	=SUL (0.125-0.5 mg/L)	=SUL (0.125-0.5 mg/L)
AUC_{0-24}/MIC	Clinical efficacy	=SUL (4 mg/L)	=SUL or CPZ (8 mg/L)	=CPZ (8 mg/L)	=SUL (4 mg/L)	=SUL (4 mg/L)
	Microbiological efficacy	=SUL or CPZ (2 mg/L)	=CPZ (2 mg/L)	=CPZ (2 mg/L)	=SUL (2 mg/L)	=SUL (2 mg/L)
		Dosing regimen of CPZ/SUL (q6h)				
%T>MIC	Clinical efficacy	=SUL (4-16 mg/L)	=SUL (8-32 mg/L)	=SUL (16-32 mg/L)	=SUL (4-16 mg/L)	=SUL (4-16 mg/L)
	Microbiological efficacy	=SUL (1-8 mg/L)	=SUL (2-16 mg/L)	=SUL (4-32 mg/L)	=SUL (1-8 mg/L)	=SUL (1-8 mg/L)
AUC_{0-24}/MIC	Clinical efficacy	=SUL (8 mg/L)	=SUL or CPZ (16 mg/L)	=CPZ (16 mg/L)	=SUL (8 mg/L)	=SUL (8 mg/L)
	Microbiological efficacy	=SUL or CPZ (4 mg/L)	=CPZ (4 mg/L)	=CPZ (4 mg/L)	=SUL (4 mg/L)	=SUL (4 mg/L)

=SUL: PK/PD cutoff based on combined PTA = PK/PD cutoff based on sulbactam

Close to SUL: PK/PD cutoff based on combined PTA was close to PK/PD cutoff based on sulbactam

=SUL or CPZ: PK/PD cutoff based on combined PTA = PK/PD cutoff based on sulbactam or cefoperazone

Close to CPZ: PK/PD cutoff based on combined PTA was close to PK/PD cutoff based on cefoperazone

=CPZ: PK/PD cutoff based on combined PTA = PK/PD cutoff based on cefoperazone

In parathesis, range of PK/PD cutoff was obtained from combined PTA. Cutoff was for cefoperazone. Infusion time was 0.5-4h.

Table S12. Effect of age on PK/PD cutoff of sulbactam

Age	Clinical efficacy					Microbiological efficacy				
(years)	T=0.5	T=1	T=2	T=3	T=4	T=0.5	T=1	T=2	T=3	T=4
18	0.5	0.5	2	4	4	0.125	0.125	0.25	0.5	1
70	4	4	8	8	8	1	2	2	2	4

T means infusion time (h). Unit of cutoff: mg/L.

PK/PD index was %T>MIC. Target for clinical and microbiological efficacy was 36.6% and 61.1%, respectively.

Dosing regimen of sulbactam was 1g(q8h)

Table S13. Effect of age on CFR of %>MIC of sulbactam

Age	Clinical efficacy					Microbiological efficacy				
(years)	T=0.5	T=1	T=2	T=3	T=4	T=0.5	T=1	T=2	T=3	T=4
18	23	26	35	46	50	8	10	13	18	26
70	57	60	64	67	68	37	40	44	48	54

T means infusion time (h). Unit of CFR: %

PK/PD index was %T>MIC. Target for clinical and microbiological efficacy was 36.6% and 61.1%, respectively.

Dosing regimen of sulbactam was 1g(q8h)

Table S14. Inclusion and exclusion criteria

No	Inclusion criteria
1	Sign an informed consent
2	Age was 18-80 years old
3	Women of childbearing age had negative results for urine pregnancy test (limit of detection of β-HCG was 25 IU/L)
4	Inpatients diagnosed with HAP or VAP caused by MDR Ab or PA sensitive or intermediate to CPZ/SUL
5	Systematic antibacterial therapy was less than 48h before enrollment. If systematic antibacterial therapy was longer than 48h, patients with unsolved infection symptoms or signs, and positive sputum culture could be enrolled
**No**	**Exclusion criteria**
1	HAP was accompanied by one of following:<br>i) Respiratory failure defined as FiO_2>35% which maintains SaO_2>90%<br>ii) Chest X-ray examination showed evidence of rapid progressive infiltration involved multilobar or porosis formation<br>iii) Severe pyemia with hypotension or/and evidences of organic dysfunction (such as shock defined as SBP < 90mmHg or DBP < 60mmHg, requiring > 4h of administration of vasopressors)
2	Patients with severe VAP diagnosed by one item of major criteria or two items of minor criteria:<br>Major criteria: i) Conscious disturbance caused by pneumoniae; ii) Septic shock; iii) Impairment of renal function (urine volume < 80ml/4h) or the elevation of serum creatinine for patients with normal renal function before; iv) Progressive decrease of oxygenation index (PaO_2/FiO_2) or pulmonary compliance, or progressive increase of air way resistance which could not be explained by non-infection factors; v) Enlarge of area of radiographic infiltration in lung > 50% within 48h.<br>Minor criteria: i) Hyperpyrexia (⩾39°C) or hypothermia (⩽36°C); ii) Radiographic infiltration in lung was multilobar or bilateral; iii) SBP < 90mmHg; iv) DBP < 60mmHg; v) Impairment of liver function after exclusion of basic liver disease and drug-induced liver injury
3	Patients with infection caused by bacteria (such as methicillin-resistant Staphylococci, Listeria and Enterococci) beyond antibacterial spectrum of CPZ/SUL
4	Patients with leucocytopenia (WBC<4.0×10^9/L) or neutrocytopenia (granulocyte < 2.0×10^9/L), or patients with immunodeficiency treated by glucocorticosteroid (prednisone 20mg/d, more than two weeks), or by immunosuppressive agents
5	Patients with combination therapy that might affect efficacy of CPZ/SUL after exclusion of vancomycin, norvancomycin, teicoplanin and antifungal agents
6	Patients with disease that might have impacts on course and evaluation of infectious process, e.g., bronchial obstruction, obstructive pneumonia, and active pulmonary malignant lesions
7	Patients with liver failure or severe bile duct obstruction. ALT and/or AST were ⩾3 times of upper normal limit
8	Patients with history of allergy to penicillins, cephalosporins, sulbactam or tazobactam
9	Pregnancy or breast-feeding women
10	Patients with hemorrhagic tendency
11	Any conditions considered by investigators that might increase the risk of patients or interfere with study results

HAP and VAP definitions:

- HAP refers to pneumonia occurred 48h after the admission, or pneumonia occurred 48h after leaving hospital for patients infected during hospitalization. VAP refers to pneumonia which occurred 24h after endotracheal intubation and mechanical ventilation, or pneumonia occurred 48h after stopping mechanical ventilation and clearing artificial airway
- Patients had progressing infiltration on chest radiograph
- One of following criteria should be met: body temperature > 37.5°C or rectal temperature > 38°C; white blood cell count >10×10^9/L or neutrophil count > 70%
- At least two of following should be met: cough, chest pain, mosist crackles in lung by auscultation or purulent sputum

Abbreviations: Ab *Acinetobacter baumannii*, ALT alanine aminotransferase, AST aspartate aminotransferase, CPZ/SUL cefoperazone/sulbactam, DBP diastolic blood pressure, HAP hospital-acquired pneumoniae, MDR multi-drug resistant, SBP systolic blood pressure, PA *Pseudomonas aeruginosa*, VAP ventilator-associated pneumonia, WBC white blood cell count

Table S15. Sampling times of cefoperazone/sulbactam

Group	Day of sampling		Pre-dose	Mid-point of infusion	After the end of infusion (h)							
					0	0.5	1	2	4	6	12	16
1	4th day	1st dose	√	√	√	√	√	√	√	√		
2	Last day	Last dose		√	√	√	√	√	√	√		√
3	Last day	Last dose		√	√	√	√	√	√	√	√	
4	1st day	1st dose			√	√	√	√	√	√		

Infusion time was 1.5-2 h

Table S16. Definition of success and failure for clinical and microbiological efficacy

Efficacy	Result	Definition
Clinical Efficacy	Success	Recovery of infectious symptoms, signs, clinical laboratory tests and X-ray test to normal or pre-infection state
	Failure#	1) Infectious symptoms, signs and laboratory test did not improve or even get worse within 72 h after drug therapy<br>2) Infectious symptoms appeared again after complete of therapy<br>3) Other antibacterial were needed for continuous therapy<br>4) Adverse drug reactions appeared which result termination of drug administration before complete of treatment course
Microbiological efficacy	Success+	1) Bacterial culture is negative after drug therapy<br>2) Patient did not have material for bacterial culture and clinical efficacy was success<br>3) Eradication of original pathogen and appearance of new pathogen without any infectious symptoms
	Failure#	1) Bacteria still exists after drug therapy<br>2) Bacterial culture turns to negative, then bacteria reappear during treatment<br>3) Bacteria are cleared. However, new bacteria appear accompanied with clinical symptoms and signs

+ Efficacy is success if the situation meets one of definitions

Efficacy is failure if the situation meets one of definitions

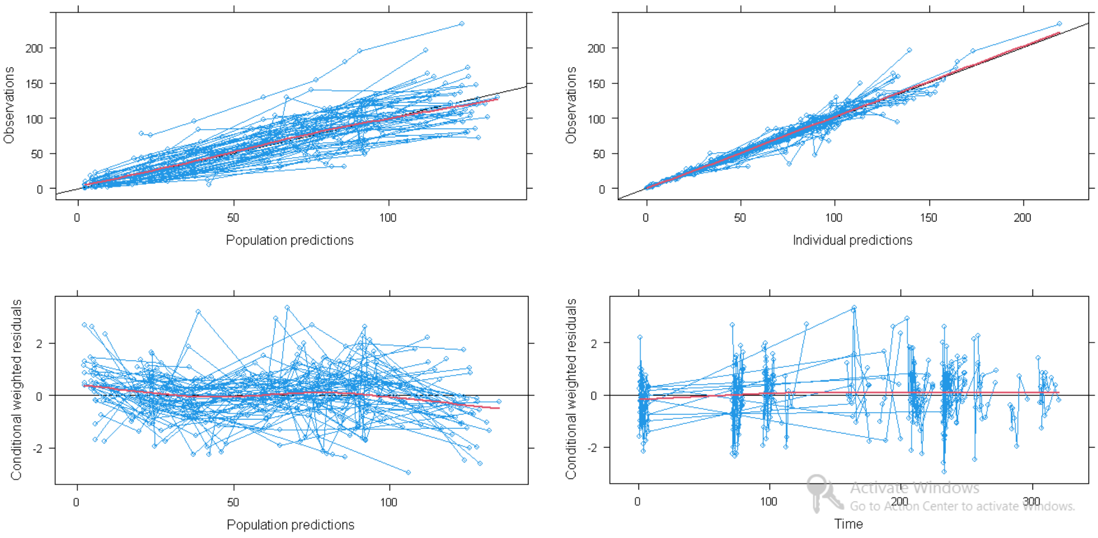

Figure S1. Goodness of fit plot for population pharmacokinetic model of cefoperazone. Blue circle: actual data. Black line: identity line (first row) or zero horizontal line (second row). Red line: locally weighted linear regression.

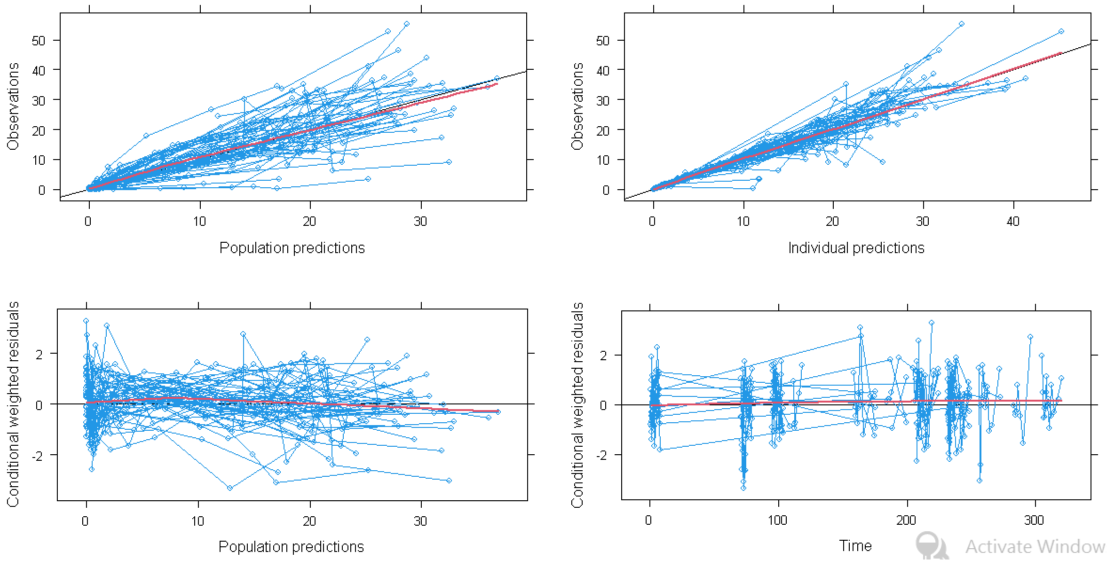

Figure S2. Goodness of fit plot for population pharmacokinetic model of sulbactam. Blue circle: actual data. Black line: identity line (first row) or zero horizontal line (second row). Red line: locally weighted linear regression.

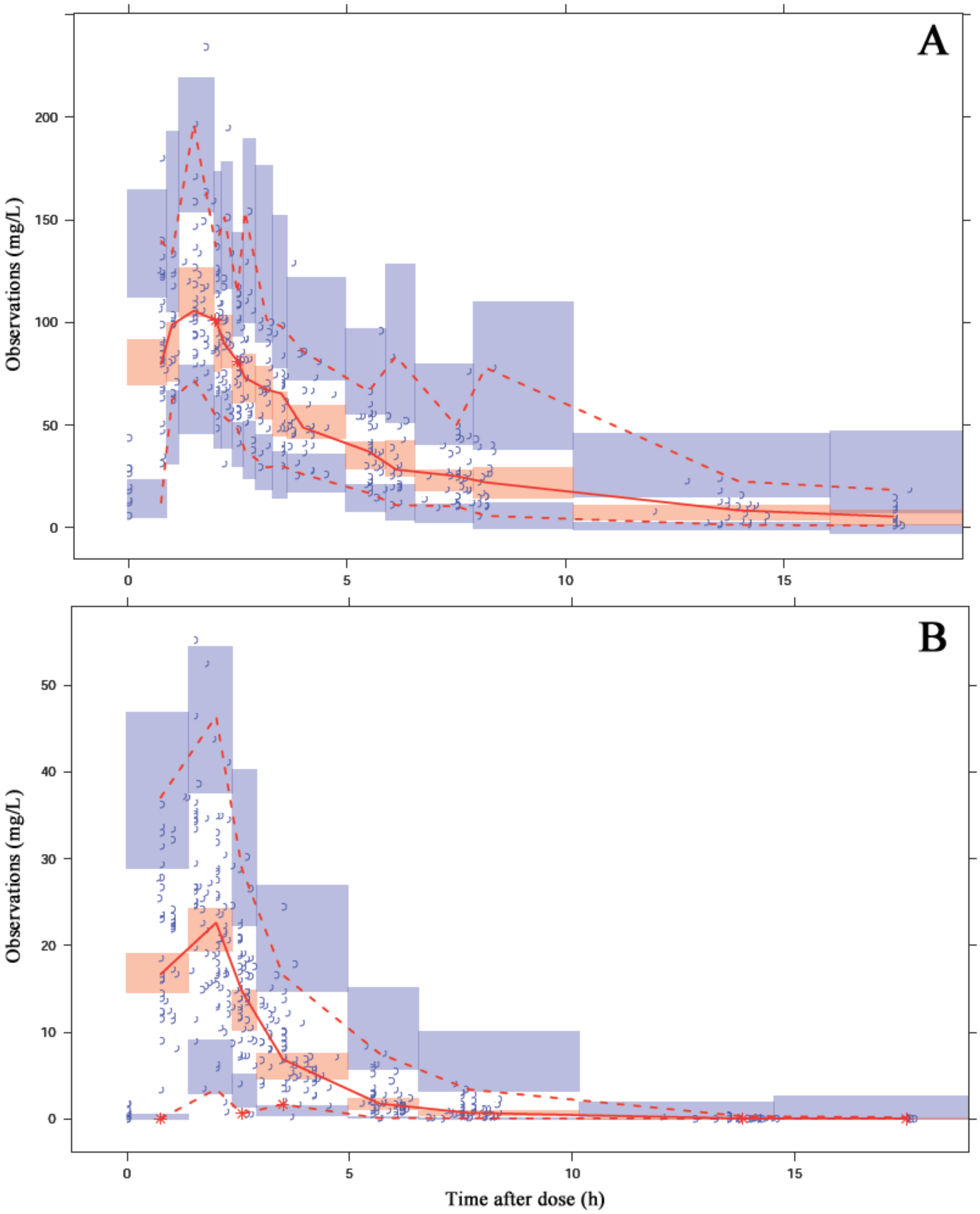

Figure S3. Visual predictive check for population pharmacokinetic model of cefoperazone (A) and sulbactam (B). Solid and dash red line mean median and 90% percentile for actual data, while red and blue area mean 95% confidence interval for median and 90% percentile obtained from simulated data.
